# Behavior of Wild Pigs toward Conspecific Carcasses: Implications for Disease Transmission in a Hot, Semiarid Climate

**DOI:** 10.1155/2023/4195199

**Published:** 2023-02-21

**Authors:** Samantha Leivers, Tyler Campbell, Michael Bodenchuk, John Tomeĉek

**Affiliations:** ^1^Department of Rangeland, Wildlife and Fisheries Management, Texas A&M University, 495 Horticulture Road, College Station, TX 77843, USA; ^2^East Foundation, 200 Concord Plaza Dr#410, San Antonio, TX 78216, USA; ^3^Wildlife Services, Animal Plant Health Inspection Service, United States Department of Agriculture, San Antonio, TX 78269, USA

## Abstract

Wild pigs (*Sus scrofa*) are a prolific, invasive species in the United States of America and act as vectors for many pathogens. An emerging pathogen of concern to the USA is African swine fever (ASF), a deadly viral disease affecting swine that is endemic to Africa and has spread to parts of Europe, Asia, and the Caribbean. ASF affects both wild and domesticated pigs and can be transmitted via several avenues, including interactions between and consumption of dead pigs by their live conspecifics. As wild pigs are considered a serious threat in the transmission of ASF, understanding the behavior of wild pigs towards their dead conspecifics is imperative when considering the transmission of ASF and other diseases in the USA. We placed camera traps at a sample of wild pig carcasses dispatched during four aerial shooting events between November, 2020, and June, 2022, at East Foundation's San Antonio Viejo Ranch, South Texas. We recorded visitation events to carcasses by live wild pigs and recorded their behavior. Furthermore, we assessed daily carcass decomposition rates by visiting carcass sites without cameras. We found no evidence of cannibalism and recorded live wild pig visitations to only 33% of carcasses before advanced stages of decomposition were reached. Carcass decomposition was rapid (2.5 to 3 days), regardless of season, and the time to the first visitation and investigation of carcasses by live conspecifics was quicker than has been recorded in Europe. We posit that active scavenger guilds at our study site, coupled with high temperatures, result in the rapid decomposition of wild pig carcasses, which reduces opportunities for live wild pigs to interact with them when compared to milder climates. We suggest additional research investigating the persistence of ASF in hot, arid climates and the interactions between live pigs and the skeletonized remains of conspecifics.

## 1. Introduction

Wild pigs (*Sus scrofa*) are a member of the Suidae family, native to Eurasia, now found on all continents except Antarctica [1]. Commonly known by many names, such as feral swine, feral pigs, wild boars, or wild hogs, wild pigs refer to the Eurasian wild boar, feral domesticated swine, and their hybridized offsprings [2]. Spanish explorers introduced wild pigs to the Americas in the 1500s [2], and today, the species' range extends across much of the United States of America (hereafter USA), Mexico, and some regions of Canada. Wild pigs present significant challenges in the USA and abroad in the form of damages to agriculture, the environment, and risks to human, wildlife, and livestock health.

Wild pigs vector and serve as reservoirs for at least 30 viral and bacterial diseases and 40 parasites [3]. These include swine brucellosis, pseudorabies, leptospirosis, tuberculosis, porcine reproductive and respiratory virus (PRRSV), hepatitis E, anthrax, toxoplasmosis, and influenza A virus [4–7]. Most of these pathogens can be transmitted to humans and nonhuman animals through direct contact with wild pigs, their scat or urine, by using feeding and watering sources that have been contaminated by wild pigs, or by consuming infected tissue from wild pigs (e.g., undercooked meat). Given that domestic swine farming is an important contributor to the economy of the USA, with pork export sales reaching nearly $7 billion USD in 2019 [8], swine zoonoses often lead to severe economic consequences due to the threat of novel pathogens to humans, a decrease in public demand for pork, culling of domestic swine, and international sanctions on the export of pork [9]. PRRSV is the most economically significant swine pathogen in the USA, resulting in estimated annual losses of $664M USD [10]. Outbreaks in both the USA and abroad of diseases currently considered eradicated in the USA domesticated swine industry (e.g., pseudorabies, brucellosis) indicate the significant economic and socioeconomic impacts of emerging pig zoonoses [9, 11–15].

An emerging viral pathogen of concern to the USA and across the globe is African swine fever (ASF). ASF is a viral disease affecting swine and is endemic to sub-Saharan Africa. In 1957, outbreaks of the disease occurred for the first time outside its endemic zone, occurring in Portugal, before spreading to France and Spain and arriving in the Caribbean in 1978. ASF was reintroduced to Europe via Georgia in 2007, before spreading to eastern and central Europe, Vietnam, Mongolia, People's Republic of China, and, most recently, Haiti, and the Dominican Republic [16]. ASF causes fever, internal bleeding, and abortion in infected swine, and causes death in 95–100% of infected swine [17]. Although it has coevolved with wild African suids, including the common warthog (*Phacochoerus africanus*) and bushpig (*Potamochoerus porcus*), ASF is not known to infect other animals or humans. The pathogen spreads via several avenues, notably contact with infected animals (including contact between domesticated swine and wild pigs), the ingestion of meat or meat products from infected animals [18], and through ticks of the genus *Ornithodoros* [17]. The economic impacts of ASF are not well understood, but research studies in Vietnam after the death/culling of 20% of the country's domestic swine population due to ASF showed severe direct and indirect economic losses among farmers, particularly medium- and large-scale farmers whose livelihoods are largely derived from swine production [14].

Currently, ASF is not present in the USA, but USDA-APHIS [19] predicts that there is a high likelihood of it entering the country via illegal entry of swine products and byproducts. Current research is also seeking to understand the ability of non-*Ornithodoros* ticks to vector the disease. Recent statistical modelling efforts indicate that the introduction of ASF to the USA would likely result in significant economic losses and job losses, due in large part to the inability to export pork [20]. The authors evaluated 2 scenarios—the “all-years” scenario, whereby the disease spreads to wild pigs and the USA is unable to eliminate the disease over the 10-year projection period, and the “2 year” scenario, which assumes that the USA gets the disease under control and reenters export markets within 2 years. Both scenarios would result in an immediate 40%–50% reduction in USA live swine prices, with the 2-year scenario predicting an economic loss of $15 billion USD and the all-year scenario predicting a loss of $50 billion USD. In addition, the all-years scenario predicts a loss of 140,000 jobs. With the high likelihood of ASF entering the USA and the severe economic and socioeconomic repercussions of its introduction, understanding the avenues through which ASF may spread throughout the USA upon its introduction is imperative for proactive management decisions.

One of the greatest concerns with the spread of ASF in the USA is that infected wild pigs will both transmit the disease to domesticated livestock and spread the disease to new, uninfected regions, as has been noted in other parts of the world [6, 9, 20]. Many pathogens carried by wild pigs are transferred through contact with infected individuals (e.g., sexual contact and nose-to-nose contact), contact with the urine or feces of infected individuals, or the consumption of infected tissues. With diseases that have high mortality rates, such as ASF, understanding the nature, timing, and frequency of interactions between live wild pigs and their dead conspecifics is an important consideration of disease epidemics, particularly when pathogen tolerance varies based on the natural environment [21–23]. The physical investigation or cannibalism of dead conspecifics could result in the transmission of disease, but the literature varies on the reported behaviors and frequency of contact between live wild pigs and the carcasses of their conspecifics. Research studies in the Czech Republic investigating ASF transmission showed that 9.8% of interactions between wild pigs and dead conspecifics during the winter months resulted in cannibalism, with the first interactions with the carcass happening after 30 days and cannibalism occurring after 70 days [24]. Similar research in Germany showed that 26% of wildlife interactions with wild pig carcasses were attributed to wild pigs, with direct contact between carcasses and live wild pigs occurring in roughly 33% of conspecific interactions [25], although no direct cannibalism was observed. On average, carcasses were approached on day 7, and direct contact with a carcass occurred on average at day 15. In these studies, data were collected in temperate oceanic or warm-summer humid continental climates [26]. Temperatures in these climates rarely exceed 20°C, providing an environment in which ASF can remain active on the landscape in tissue and bone marrow for months to years [22, 23]. As such, these interactions represent a risk of ASF transmission from dead wild pigs to living conspecifics.

However, several factors may influence interactions, including cannibalism, between wild pigs and their dead conspecifics. For example, carcasses decompose more quickly on the landscape in hot climates [27, 28], potentially providing fewer opportunities for wild pigs to interact with them. Indeed, Cukor et al. [24] noted that carcasses in their study decomposed extremely slowly due to the cold climate and were not colonized by insects, thus increasing the temporal availability of the carcasses on the landscape and allowing for potential interactions with live conspecifics. Another consideration is the time of year at which the research is conducted. Cukor et al. [24] posited that cannibalism may have occurred due to the lack of alternative protein sources in the environment at the end of the cool season, although research by Probst et al. [25] found no evidence of cannibalism in either the warm or cool season in a similar climate. Further research investigating wild pig interactions with their conspecifics across different climates and seasons is warranted.

Texas hosts one of the largest and most persistent populations of wild pigs in the USA, with an estimated population of at least 3.6 million wild pigs residing in Texas as of 2019 and an annual growth rate of approximately 0.32 [29]. Wild pigs in Texas have some of the highest rates of toxoplasmosis, pseudorabies, and trichinellosis in the country [30]. In addition to their large numbers, South Texas has one of the warmest climates in the USA [31], providing an ideal scenario to investigate interactions between live and dead wild pigs in hotter, more-arid climates and how this might influence disease transmission. As ASF is considered likely to enter the USA via the illegal transport of swine products, Texas' role as an international border state means that understanding the potential for ASF to spread in the state is important in planning for and mitigating the introduction of ASF. In this study, we investigate the behavior of wild pigs towards their dead conspecifics and examine rates of carcass decomposition during the cool and warm seasons in Southern Texas. We discuss our findings in terms of their implications for disease transmission, especially ASF, in hot, semiarid climates.

## 2. Materials and Methods

### 2.1. Study Site

**Figure 1:**
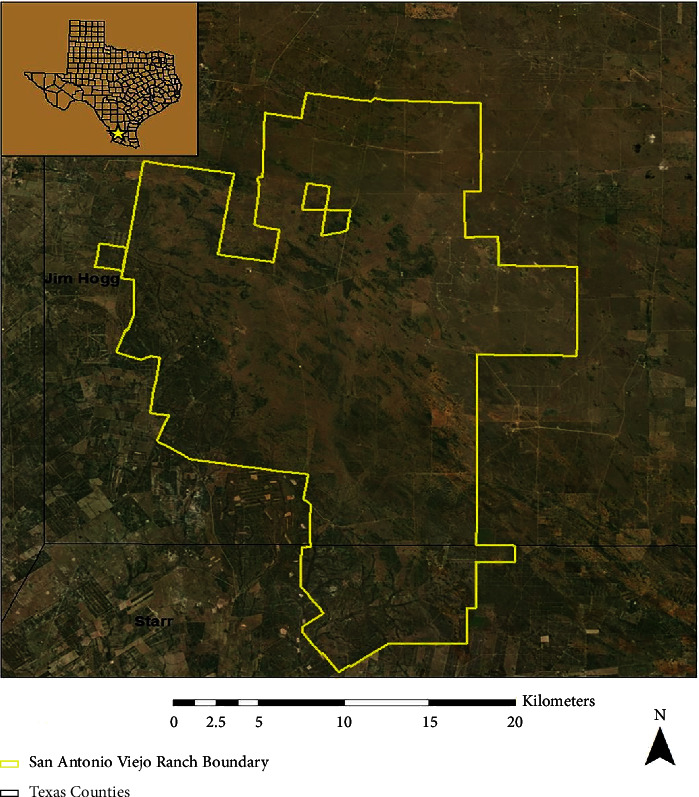
East Foundation's San Antonio Viejo Ranch, in Jim Hogg and Starr counties of Southern Texas (taken with permission from reference [32]).

We conducted this study on the East Foundation's 61,000 ha San Antonio Viejo Ranch (SAVR) in Jim Hogg and Starr counties in South Texas (Figure 1). The East Foundations Ranches are managed as a living laboratory to promote the advancement of land stewardship through ranching, science, and education. The area is dominated by shrub savannas, primarily composed of honey mesquite (*Prosopis glandulosa*), prickly pear (*Opuntia* spp.), cat-claw acacia (*Acacia greggii*), blackbrush (*Acacia rigidula*), whitebrush (*Aloysia gratissima*), and granjen˜o (*Celtis palida*), with early to mid-successional grasses, including three-awns (*Aristida* spp.), little bluestem (*Schizachyrium scoparium*), and windmill grasses (*Chloris* spp.). SAVR has a hot, semiarid climate [26]. The 30-year normal mean temperature for the region is 22.2–23.9°C (max temperature 31.1–33.3°C) with an annual mean precipitation of 50.8–60.96 cm [31].

### 2.2. Carcasses

We collected wild pig carcasses opportunistically during biannual wild pig control efforts undertaken by USDA-APHIS-Wildlife Services (hereafter WS) during the course of routine operations to reduce wild pig damage on SAVR. Aerial gunning events took place during both the cool season (17–19 November, 2020, and 8–11 November, 2021) and the warm season (24–26 May, 2021, and 6–9 June, 2022). WS aerial teams located wild pigs from a helicopter and euthanized animals using a shotgun and nontoxic #2 (6.86 mm diameter) buckshot ammunition in accordance with routine WS aerial operations procedures. Once a wild pig, or a sounder of wild pigs, had been euthanized the helicopter crew provided ground crews with GPS coordinates to the site of the carcass(es) and the number of wild pigs euthanized via radio. The date and time of each euthanasia event were also recorded. Upon arrival at a carrion site, the ground crew assigned a site ID and a pig ID to each euthanized pig at the site. Ground crews confirmed the number of pigs at the site, the age, sex, and color/markings of all pigs at the site, and weighed each pig. Ground crews dragged carcasses located in thick vegetation (e.g., under a bush) to the closest area with vegetation sufficiently open to allow for data collection. Carcasses were also moved from locations that would impede normal ranch operations (i.e., directly in front of a gate). If more than one wild pig was present at the site, carcasses were laid next to each other. Ground crews collected the exact site GPS coordinates of the carcass (es), recorded the date and time of the site visit, and took a photograph of the carcass (es).

### 2.3. Carrion Sites

We assigned 137 carrion sites to either a trail camera condition to determine wild pig interactions with dead conspecifics (*N* = 88: 62 cool season sites, 26 warm season sites) or a control condition to determine wild pig carcass depletion through decomposition and scavenging (*N* = 49: 27 cool season sites, 22 warm season sites). Sites from the June 6 to 9, 2022 gunning event were only included in the control condition, pending final analysis of camera photos. We considered carcass decomposition and depletion at control sites to be a true reflection of these metrics on the landscape, as scavenger activity would not be potentially influenced by the presence of trail cameras [33] and, if moved from the original site, remains could be tracked and decomposition scores continued to be assigned. Carcasses at control sites were visually inspected once per day until six days after euthanasia (unless the carcass disappeared or was fully depleted) and assigned a decomposition score between 1 and5 (Table 1). This scale is based on the total body score (TBS) used for humans but simplified for camera use [34, 35]. Several additional carrion sites were not visited due to timing constraints, difficulty accessing the site, and/or because they were being used for another experiment and thus were not included in any analyses.

Carcasses at trail camera sites were positioned with the ventral side facing the trail camera, approximately 5 meters from the camera. This distance increased as the number of carcasses at the site increased in order to fit all carcasses in the frame. Trail cameras were attached to existing vegetation (e.g., tree trunks), fence posts, or where this was not possible, attached to steel T-posts driven at the site by researchers. Loose vegetation was removed from in front of the camera to prevent false triggers and provide a clear view of the carcass(es). We used both HyperFire 2 Professional HP2X and HyperFire Professional PC900 IR trail cameras with external data cards. Trail cameras were set to high sensitivity and were programmed to take a burst of three photographs, three seconds apart, when triggered, with a quiet period of one minute between subsequent triggers. Cameras were active throughout the full 24-hour period to capture both day and night-time visits by wildlife.

Photographs from trail cameras were downloaded once, after the carcass had been depleted or dragged from sight. None of the data cards were full prior to carcass depletion or removal, so we determined that we had no loss of data due to memory limitations. Photographs were transferred to a desktop computer and uploaded to an online file sharing platform for processing. Photographs were examined for the presence of live wild pigs at each carrion site from the time of the first trigger until the carcass(es) was/were depleted (i.e., no bones, viscera or other remains were in view) or was removed from the camera's frame by humans or animals and did not return to the frame. Data were recorded in a relational database created using FileMaker Pro. For each photograph in which live wild pigs were present, we recorded the following: site ID, time and date of observation, number of live wild pigs, decomposition score of carrion (1–5, unknown if view was obscured), time of day (morning: daylight to 11:59, afternoon: 12:00 to darkness, night: all infrared images), and all behaviors performed, including whether the carrion was observed, investigated, or consumed (Table 2).

Daily mean and maximum temperatures (°C) and precipitation (cm) at SAV headquarters for 7 days of each of the three aerial gunning events (the first day of aerial gunning plus six days later) were downloaded from PRISM Climate Data [31].

## 3. Results

### 3.1. Carcass Decomposition

There were a total of 66 wild pig carcasses (cool season: *N* *=* 37, Max = 3, warm season: *N* = 29, Max = 5) at 49 control carrion sites (cool season: *N* = 27, warm season: *N* = 22). The mode number of carcasses at each control carrion site was 1 for both the cool season and warm season. The mean mass of carrion at each control site across seasons was 61.30 kg (S.D = 36.67) but varied between seasons (cool season: *x̄* *=* 63.44 kg, S.D = 31.07, warm season: *x̄* = 58.77 kg, S.D = 43.00), although this difference was not significant (Mann−Whitney *U* test, *Z* = 0.88, *p* > 0.05). Twenty-nine (43.9%) of the carcasses were male and 37 (56.1%) were female, and 5 of the 66 control sites (7.6%) had carcasses of both males and females. Eleven carcasses were removed from their respective carrion sites by humans or wildlife and could not be recovered for decomposition assessments, and a further 8 carcasses were dropped from further analysis due to a lack of data (i.e., data collection stopped prior to six days post-euthanasia and before reaching a decomposition score of 5) or data entry errors. Of the remaining 47 carcasses, 45 (95.74%) reached a decomposition score of 5 within 6 days of euthanasia (cool season sites: 25/27, 92.59%; warm season sites: 22/22, 100%). The median time taken for a carcass to reach a decomposition score of 5 was 3 days for the warm season and 2.5 days for the cool season.  Table 3 presents the daily mean and maximum temperatures for each aerial gunning event.

### 3.2. Wild Pig Visitations

Data from 29 trail camera sites were excluded from analysis due to incomplete data processing (*N* = 16), camera placement/technical errors (*N* = 6), or carcasses being removed from the view of the trail camera by humans or wildlife within 24 hours of the time of euthanasia (*N* = 7). Therefore, we analyzed data from 59 trail camera sites (cold season: *N* = 42, warm season: *N* = 17), for a total of 38365 photographs (up until carcasses were depleted or were removed from view). Data were collected for between 2 and 9 days for a total of 232 traps days (cool season: 170 days, warm season: 62 days) with the mode number of trap days being 3. There were a total number of 91 wild pig carcasses (cool season: *N* *=* 59, Max = 6, warm season: *N* = 32, Max = 5) at the 59 trail camera sites. The mode number of carcasses at each camera site was 1 for both the cool season and warm season. Thirty-five (38.5%) of the carcasses were male and 56 (61.5%) were female, and 9 of the 59 trail camera sites (15.3%) had carcasses of both males and females. The mean mass of carrion at each camera site across seasons was 71.66 kg (S.D = 43.21) but varied between seasons (cool season: *x̄* *=* 64.93 kg, S.D = 37.85, warm season: *x̄* = 87.91 kg, S.D = 51.72), although this difference was not significant (Mann−Whitney *U* test, *Z* = −1.55, *p* > 0.05).

**Figure 2:**
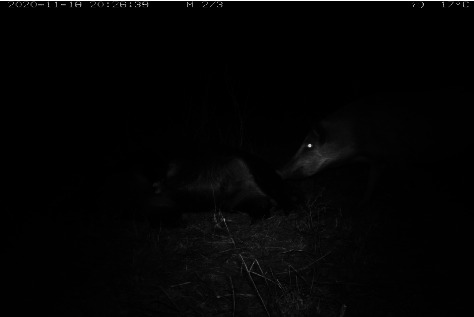
A wild pig investigates the carcass of a conspecific killed during an aerial gunning event at East Foundation's San Antonio Viejo Ranch, South Texas, in November, 2020.

**Figure 3:**
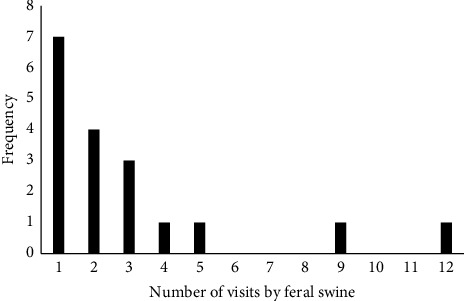
Frequency of wild pig visitations to 18 wild pig carrion sites as recorded by trail cameras during 3 aerial gunning events held in November 2020, May, 2021, and November, 2021, at East Foundation's San Antonio Viejo Ranch, South Texas.

Wild pigs were documented visiting 18 of the 59 carrion sites on a least 1 occasion, for a total of 54 visitation events across seasons (Figure 2). Sixteen of 42 cool-season sites (38.1%) were visited for a total of 51 visitations, and 2 of 17 warm-season sites (11.84%) were visited for a total of 3 visitations. Most sites that were visited by wild pigs were visited on only 1 occasion (Figure 3). The mean time taken for wild pigs to first visit a site from the time of euthanasia was 42.3 hours (S.D = 17.1 hours, min = 8.7 hours, max = 66.5 hours) in the cool season and 29.04 hours (S.D = 30.8 hours, min = 7.6 hours, max = 51.2 hours) in the warm season. Six of the visited carrion sites had male carcasses, 10 had female carcasses, and 2 had both male and female. There was no significant effect of carcass sex on visitation by wild pigs (two sites with both sexes excluded from analysis, chi-squared test with Yates correction, *X*^*2*^ = 0.64, *p* > 0.05). There was no effect of carrion mass on whether a carrion site was visited by live wild pigs (Mann–Whitney *U* test, *Z* = −0.01, *p* > 0.05).

**Figure 4:**
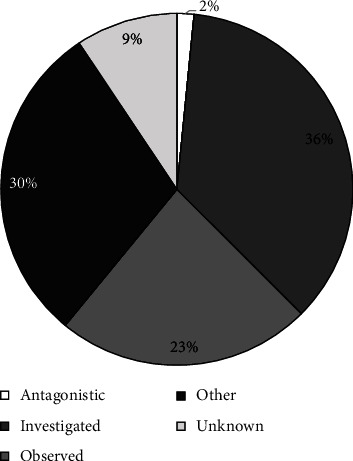
Behaviors observed in wild pigs towards their dead conspecifics as recorded by trail cameras at 18 carrion sites created via 3 aerial gunning events held in November, 2020, May, 2021, and November, 2021, at East Foundation's San Antonio Viejo Ranch, South Texas.

**Figure 5:**
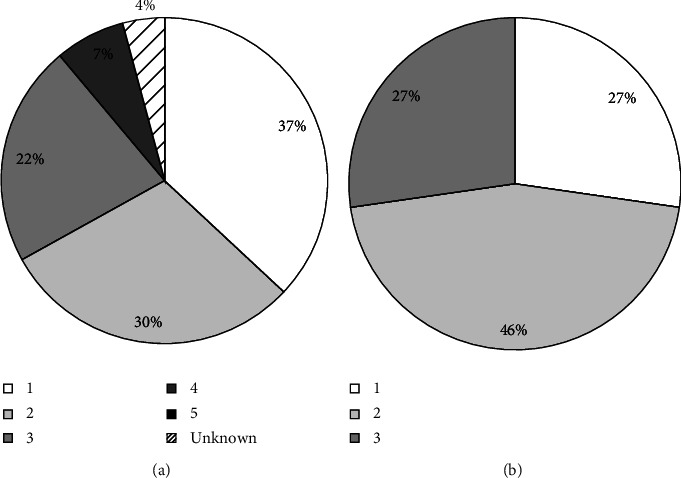
Percentage of (a) total interactions and (b) total investigative interactions of wild pigs towards conspecific carcasses as measured by carcass decomposition score as recorded by trail cameras at 20 carrion sites created via 3 aerial gunning events held in November, 2020, May, 2021, and November, 2021, at East Foundation's San Antonio Viejo Ranch, South Texas. “1” = fresh; has not been scavenged on or decomposed yet “5” = completely scavenged; flesh consumed or decomposed; mostly bones, “Unknown” = carcass obscured from view.

We recorded 64 behavioral events during the 54 visitation events (Figure 4). The most common behavior recorded was “investigated,” followed by “other” and then “observed.” Notably, there were no instances of cannibalism. In only 4 of 54 visitation events (7.4%), were there more than 1 wild pig present in the photograph (max = 3). Most wild pig visitation events took place during the night (94.4%), with 3.7% visitations during the morning and 1.9% in the afternoon. Most visitation events occurred when carcasses were at a decomposition score of 1 (Figure 5(a)). There were 23 recorded instances of wild pigs investigating carcasses at 9 of the 18 visited carrion sites (50.00%), all of which took place during the cool season. The mean time taken by a wild pig to first investigate a carcass from the time of euthanasia was 49.0 hours (S.D = 19.3 hours, min = 27.30 hours, max = 87.1 hours), and most investigative interactions took place when carcasses were at a decomposition score of 2 (Figure 5(b)).

## 4. Discussion

Our study had several key findings, including: (1) no observations of cannibalism between wild pigs, (2) only 33% of carrion sites were visited by live wild pigs before advanced states of decomposition were reached, (3) carcass decomposition was rapid, regardless of season, and (4) the time to first visitation and investigation of wild pig carcasses by live conspecifics was considerably quicker in Texas than has been recorded in Europe.

It is notable that there was no evidence of consumption of dead conspecifics by wild pigs, as this has been observed in wild pigs in Europe and is recognized as a transmission route for ASF [24]. Ingestion of infected tissues is a very effective way of transmitting ASF, as the virus is present in high loads in the tissues and body fluids of infected animals [24]. Nevertheless, physical investigation of an infected carcass can still lead to the transmission of disease, either through exposure to infected bodily fluids and tissue, or disease vectors such as ticks. The transmission of ASF has been linked to ticks of the genus *Ornithodoros* in Africa and the Mediterranean, and laboratory studies indicate that several North and Central American species have the potential to transmit ASF (*O. coriaceus*, *O. turicata*, *O. parkeri*, and *O. puertoricensis*) [17, 36, 37]. Reports, literature reviews, and field studies show that *O. turicata* occurs throughout many areas of Texas, including our study site [38]. This represents an additional avenue of virus transmission during the conspecific investigation of dead carcasses. Indeed, carcass investigation was the most commonly recorded behavior of wild pigs towards dead conspecifics (and happened at half of carrion sites visited), thus creating the opportunity for disease transmission through both interaction with infected tissues and transmission of potentially infected ticks. Our findings suggest that the behavior of wild pigs towards conspecifics in South Texas presents the opportunity for ASF transmission, although potentially not at the rates one might see in Europe, where cannibalism has been observed.

An additional consideration when determining disease transmission potential is the rate of exposure of live wild pigs to their dead conspecifics. Unlike previous observations in Europe, where the majority of carcasses were visited, we found that live wild pigs visited only 33% of the carrion sites created via aerial shooting efforts. This is very likely due to the active scavenger guild and invertebrate communities found at the study site, resulting in quick depletion and decomposition of carcasses. Visual assessment of carcass decomposition throughout the study period showed a rapid onset of significant invertebrate activity, with larval activity observed within roughly 24 hours of death and carcasses achieving the highest score of decomposition after 2.5 to 3 days. Camera trap photographs showed that several obligate and facultative scavenger species—including turkey vultures (*Cathartes aura*), black vultures (*Coragyps atratus*), and coyotes (*Canis latrans*)—actively scavenged wild pig carcasses, which can contribute to faster rates of decomposition compared to when vertebrate scavengers are excluded [39]. Scavenger activity is an important factor in understanding disease transmission [40–42], and maintaining and supporting scavenger communities on this landscape may significantly contribute to reducing the probability of disease transmission, including ASF, between live wild pigs and dead conspecifics by removing infected materials from the landscape before live wild pigs are able to come into contact with them.

Another factor that likely affects the rate of exposure of live wild pigs to their dead conspecifics is the density of wild pigs on the landscape. Cukor et al. [24] indicated that their study sites in the Czech Republic, where cannibalism was observed, had high wild pig densities (2.98–13.78 individuals/mile^2^). Although we do not know the density of wild pigs at our study site in South Texas, density estimates on other properties in South Texas are high, varying from 6.99 individuals/mile^2^ [43] to 23.32 individuals/mile^2^ [44], although these estimates are likely outdated based on projected population growth rates in Texas [29]. Nevertheless, it is worth noting that the time to carcass discovery and rates of interaction between live wild pigs and their dead conspecifics may vary across the landscape, particularly in states such as Texas whereby a vast majority of land is privately owned and property-level wild pig control measures are at the discretion of the landowner.

Wild pig carcasses decomposed to an advanced state within 3 days on the landscape, with little difference between seasons. Although higher temperatures are associated with increased rates of decomposition [27, 28], wild pig carcasses tended to decompose slightly more quickly during the cool season than the warm season. This may be due in part to the average wild pig carcass mass being larger during the warm season shooting events, but it may also be attributed to a generally warm winter climate as temperatures did not fall below a mean of 17°C during either cool season aerial shooting event. However, although the temporal availability of carrion did not differ between the seasons, there was seasonal variation in wild pig visitations to carrion sites, with wild pigs more likely to visit carrion sites in the cool season than the warm season. Indeed, all instances of investigative behavior by wild pigs towards dead conspecifics (i.e., scenarios in which diseases could be directly transmitted) occurred during the cool season. This finding may have important management implications, as land managers may be able to mitigate the transmission of diseases from dead to live wild pigs by undertaking wild pig removal efforts in the warm season.

A potential argument against the ecological validity of our study is that aerial shooting could influence wild pig behavior in the short term, and thus we may not be observing ecologically relevant or natural behavior in live wild pigs. Indeed, this may be of particular concern due to the limited period of time that carcasses are available for interactions on this landscape due to the rapid rates of decomposition. However, previous studies show little evidence that aerial shooting results in lasting behavioral changes in surviving wild pigs [45], including those conducted within our study system [44]. While the mean distance travelled by wild pigs during an aerial shooting event was greater than the mean distance travelled both before and after aerial shooting, home range sizes did not differ before and after aerial shooting, suggesting a minimal influence of aerial gunning on the behavior of surviving wild pigs.

One limitation of our study is that we stopped carcass decomposition assessments once carcasses reached a decomposition score of 5, whereby all flesh had been consumed or decomposed. However, skin and bones remained on the landscape after this time, which could allow for the potential transmission of ASF. ASF has excellent resilience to natural environments [21–23], and ASF in bone marrow can remain active for months to years based on environmental conditions [21]. ASF resilience has mostly been studied at lower temperatures, but ASF is known to become inactive after less than 2 hours at high temperatures (56°C) in laboratory settings [23, 46] and after 11–22 days at temperatures comparable to those we report during the June 2022 aerial gunning event (i.e., 37°C). Continued research on ASF resilience in different carcass components (i.e., flesh, skin, bones) in natural-occurring, hot climates is warranted to further understand its persistence and potential for transmission in the environment. It is especially important to understand the role that invertebrate scavengers have on the persistence of the virus, as invertebrate activity can significantly increase internal carcass temperature and thus the suitability of the environment for the virus [47–49]. As carcasses and bones were often dragged from the view of the trail cameras by wildlife (namely coyotes) in our study, it was not possible to observe any interactions—if indeed there were any—between skeletonized remains and live wild pigs. This is an important avenue for future work and may be achieved through tethering carcasses in place. However, whether tethering the carcass would influence the behavior of scavengers, scavenger guild composition, and carcass utilization is worth consideration [50–52].

Our study identified possible pathways of disease transmission, including the potential for ASF transmission, from wild pig carcasses to live conspecifics in a hot, semiarid climate. However, we posit that rates of disease transmission between dead and live wild pigs may be lower than observed in Europe due to the rapid decomposition of carrion on the landscape (primarily due to active vertebrate and invertebrate scavenger communities) and the lack of evidence of cannibalism. Seasonal variation in the investigative behavior of wild pigs towards their dead conspecifics suggests that timing wild pig eradication efforts to take place during the warm season may reduce the chances of disease transmission from dead to live wild pigs.

## Figures and Tables

**Table 1:** Wild pig decomposition scale used to determine decomposition states of wild pig carcasses after 4 aerial wild pig gunning events held in November, 2020, May, 2021, November, 2021, and June, 2022, at East Foundation's San Antonio Viejo Ranch, South Texas.

1	Fresh: has not been scavenged on or decomposed yet
2	Mildly scavenged: some skin slippage, discoloration
3	Partially scavenged: muscles and viscera exposed and/or partially consumed
4	Mostly scavenged: showing bones; some flesh left
5	Completely scavenged: flesh consumed or decomposed; mostly bones

**Table 2:** Ethograms were used to quantify the behavior of live wild pigs towards their dead conspecifics after 3 aerial wild pig gunning events held in November, 2020, May, 2021, and November, 2021, at the East Foundation's San Antonio Viejo Ranch, South Texas.

Behavior	Definition
Antagonistic	Applicable when more than one wild pig is present; includes both submissive and aggressive behaviors, including chasing, fleeing from, or biting a conspecific
Consumed	Wild pig is observed with open muzzle touching carcass, or touching viscera/bones/flesh/etc., around the carcass, or with viscera/bones/flesh/etc., in the mouth
Investigated	Wild pig is observed with muzzle lowered to carcass or viscera/bones/flesh/etc., with mouth closed
Observed	Wild pigs are observed standing or moving with their heads directed towards the carcass
Unknown	No clear view of wild pig to discern the behavior
Other	Other behavior not listed above

**Table 3:** Mean (*x̄*) and maximum daily temperature (°C) and precipitation (cm) during 3 aerial wild pig gunning events at East Foundation's San Antonio Viejo Ranch, South Texas. Dates during which wild pigs were euthanized are italicized.

Season	Date	*x̄* (°C)	Maximum (°C)	Precipitation (cm)
Cool	*11/17/2020*	18.72	26.17	0
	*11/18/2020*	17.78	25.00	0
	*11/19/2020*	18.28	27.06	0
	11/20/2020	19.00	27.83	0
	11/21/2020	19.72	28.44	0
	11/22/2020	22.06	28.89	0
	11/23/2020	21.00	27.22	0

Warm	*5/24/2021*	27.06	32.17	0
	*5/25/2021*	27.33	32.56	0
	*5/26/2021*	28.67	33.78	0
	5/27/2021	28.78	34.17	0
	5/28/2021	29.00	34.44	0
	5/29/2021	28.67	34.11	0.10
	5/30/2021	24.28	29.39	0.36

Cool	*11/9/2021*	19.44	27.89	0
	*11/10/2021*	20.94	27.44	0
	*11/11/2021*	23.00	29.06	0
	11/12/2021	21.28	28.39	0
	11/13/2021	22.00	28.78	0
	11/14/2021	17.11	24.61	0
	11/15/2021	19.00	28.28	0

Warm	*6/6/2022*	29.67	37.33	0
	*6/7/2022*	31.22	38.22	0
	*6/8/2022*	31.06	38.17	0
	*6/9/2022*	31.00	37.11	0
	6/10/2022	30.72	36.67	0
	6/11/2022	31.39	38.56	0
	6/12/2022	32.11	39.56	0

## Data Availability

The data used to support the findings of this study are available from the authors upon request.
